# Minimal invasive treatment of ureteropelvic junction obstruction in low volume pelvis: A comparative study of endopyelotomy and laparoscopic nondismembered pyeloplasty

**DOI:** 10.4103/0970-1591.45540

**Published:** 2009

**Authors:** Pratipal Singh, Paresh Jain, Anand Dharaskar, Anil Mandhani, Deepak Dubey, Rakesh Kapoor, Anant Kumar, Aneesh Srivastava

**Affiliations:** Department of Urology, Sanjay Gandhi Post Graduate Institute of Medical Sciences, Lucknow, Uttar Pradesh, India

**Keywords:** Endopyelotomy, laparoscopy, pelviureteric junction obstruction, pyeloplasty

## Abstract

**Objective:**

To evaluate the role of nondismembered laparoscopic pyeloplasty and percutaneous endopyelotomy for ureteropelvic junction obstruction (UPJO) with low volume renal pelvis.

**Material and Methods:**

Retrospective acquired data of 34 patients of laparoscopic nondismembered pyeloplasty was compared with 26 patients of UPJO with pelvic volume less than 50 ml undergoing antegrade endopyelotomy and analyzed for clinical parameters, operative outcomes and success of procedures. All patients were followed up clinically and with diuretic renogram at regular intervals.

**Results:**

Mean age, renal pelvic volume and preoperative glomerular filtration rate (GFR) was 25 years, 43.6 ml and 42.5 ml/min, respectively in endopyelotomy group and 21 years, 34.4 ml and 39.9 ml/min, respectively in laparoscopic pyeloplasty group. Mean operative time, postoperative analgesic requirement and mean hospital stay was 100min, 250 mg and 4 days, respectively in endopyelotomy group and 210 min, 300 mg and 4 days, respectively in laparoscopic pyeloplasty group. Only operative time was significantly different between two groups (*P* < 0.05). Mean follow-up was 36 and 39 months and success rates were 91.2% and 88.8% in laparoscopy and endopyelotomy group, respectively (*P* < 0.05). No significant complication was seen in endopyelotomy group while two patients had hematuria (one requiring blood transfusion) and three had increased drain output for more than 3 days in laparoscopy group.

**Conclusion:**

Percutaneous endopyelotomy is associated with significantly less operative time and postoperative complication rate and provides equivalent success in comparison to nondismembered laparoscopic pyeloplasty in patients with UPJO and low volume pelvis. It can be a preferred minimally invasive treatment modality for such patients.

## INTRODUCTION

For the last few decades, open pyeloplasty has been the gold standard of surgical treatment for ureteropelvic junction (UPJ) obstruction with long-term success rate exceeding 90%. The significant morbidity of flank incision, long convalescence and prolonged hospital stay with open pyeloplasty led to development of minimally invasive treatment options like endopyelotomy and laparoscopic pyeloplasty.[1–6]

Endopyelotomy is associated with lower morbidity however, it has success rate 15 to 20% lower than that of open pyeloplasty.[1,2] Laparoscopic pyeloplasty minimizes the morbidity associated with open pyeloplasty and provides the equivalent success rate.[5,6] With widespread acceptability of laparoscopic pyeloplasty, the number of nondismembered pyeloplasty has increased proportionately initially specially in patients in small renal pelvis and probably also due to less amount suturing needed in nondismembered pyeloplasty. Kaouk *et al*. reviewed 376 laparoscopic pyeloplasties few years back and 116 of these were nondismembered.[7] Results of laparoscopic nondismembered pyeloplasty were not encouraging when compared to dismembered pyeloplasty and owing to these poor outcomes nondismembered pyeloplasty have fallen out of favor.[8]

We compared the results of nondismembered laparoscopic pyeloplasty with historical group of percutaneous endopyelotomy to define the role of latter amongst the two minimally invasive options in patients of UPJ obstruction with low volume renal pelvis.

## MATERIALS AND METHODS

From January 2002 to November 2006 185 (186 units) consecutive patients with primary (n=182) or secondary (n=4) UPJ obstruction underwent laparoscopic pyeloplasty at our institute. 34 patients among these had pelvic capacity less than 50 cubic cm and were compared with similar 26 patients of antegrade endopyelotomy done between March 2001 and July 2003. The diagnosis was made by ultrasonography (USG), intravenous urography (IVU) and diuretic renogram (99mTc99 DTPA). Retrograde pyelogram was done in cases with poorly visualized units on IVU in laparoscopy group and in all the cases of endopyelotomy group. No patient underwent renal Doppler study or computed tomographic (CT) angiography to identify crossing vessel at pelvi-uretaric junction (PUJ) prior to endopyelotomy due to cost consideration. A single operator measured the renal pelvic volume using USG. Patients with pelvic volume of less than 50 cubic cm were included in the study and patients with azotemia, infected hydronephrosis and poorly functioning kidney (glomerular filtration rate (GFR) below 25 ml/min) were excluded. The preoperative assessment for the vessels crossing the UPJ was not performed.

For antegrade endopyelotomy a posterior middle calyx was accessed and ureteropelvic junction was cut laterally down to fat using cold knife in 19 and hot knife in 6 cases. The incised area was intubated with a 14/7 Fr endopyelotomy stent (Microvasive, Natick, Mass) or 6/26 double J stent (Devon, India). Stent was left in situ for 2 to 6 weeks. Percutaneous nephrostomy was removed as soon urine was clear followed by removal of urethral catheter.

In the laparoscopy group, transperitoneal nondismembered pyeloplasty was done using standard three port technique and 4/0 polyglactin suture on round body needle was used for free hand interrupted suturing. 6/26 double J stent (Devon, India) was placed in all patients. Foley Y-V plasty was done in 26 patients and Fenger pyeloplasty was done in 8 patients. Urethral catheter was removed on first or second postoperative day and drain tube was removed once the drainage reduced to less than 25 ml in 24 hours. The double J stent was removed after 6 weeks. Both procedures were performed by the same group of surgeons.

Failed cases were managed by open dismembered pyeloplasty. Number of blood units transfused, mean operative time, analgesic requirement, and mean hospital stay were recorded.

All patients were followed up with clinically and diuretic renogram at 3, 6, and 12 months. Success was defined as disappearance of symptoms and improvement in drainage on diuretic renogram while failure was defined as persistence of symptoms and or no improvement in the drainage pattern on diuretic renogram (t½ < 15 min).

Student's t-test was used to analyze the significance of difference between the group and P value of less than 0.05 was taken as significant.

## RESULTS

The demographic parameters, mean operative time, postoperative analgesic requirement, hospitalization period and GFR of the affected kidney for both the procedures are summarized in Table 1. The mean operative time was significantly shorter in the endopyelotomy group than laparoscopy group (*P*<0.01).

**Table 1:** Comparison of patients undergoing endopyelotomy and laparoscopy

Parameter	Endopyelotomy (*n*=26)	Laparoscopic nondismembered pyeloplasty (*n*=34)	*P* value
Age [mean ± SD (range)]	25 ± 8 (8-43)	21 ± 14 (14-65)	>0.05
Sex ratio (M:F)	15 : 11	25 : 9	
Mean (± SD) analgesic requirement (Tramadol in mg)	250 ± 25	300 ± 50	>0.05
Mean (± SD) pelvic volume (ml)	43.6 ± 6.0	34.4 ± 12.1	>0.05
Mean (± SD) GFR (ml/min)	42.5 ± 15	39.9 ± 18	>0.05
Mean (± SD) operative time (min)	100 ± 20	210 ± 45	<0.01
Mean (± SD) Hospital stay (days)	4 ± 1	4 ± 3	>0.05

Mean follow up was 36 and 39 months in laparoscopy and endopyelotomy group, respectively (*P* > 0.05). Success rates were statistically similar for laparoscopic surgery group (91.2%) and for endopyelotomy (88.5%) (*P* > 0.05). In endopyelotomy group, out of 3 failed cases, 1 had vessels crossing UPJ. In laparoscopic pyeloplasty group, no patient had vessels crossing UPJ. Concomitant stone removal was done in one case in endopyelotomy group and in 3 cases in laparoscopic pyeloplasty group with 100% stone free rate in both the groups.

In laparoscopy group, two patients had hematuria in immediate postoperative period and one required blood transfusion but none of the patient in endopyelotomy group had hematuria. In laparoscopy group, three patients had increased drain output which prolonged the hospital stay to 7 days. The reasons for increased drainage were double J stent up migration in one while hematuria and clot colic in two patients. Patient with stent up migration required ureteroscopic repositioning of double J stent while others were managed conservatively.

## DISCUSSION

Endopyelotomy has become a reasonable alternative to open surgery for the treatment of ureteropelvic junction obstruction. It is less invasive, has fewer functional and aesthetic sequalae than open pyeloplasty, and does not compromise outcome of open surgery if that becomes essential. Endopyelotomy is based on the concept that re-growth of the incised ureter occurs in a non-obstructing fashion around an indwelling stent usually of standard size.[9] Long term follow up studies demonstrated that endopyelotomy should be the initial treatment of choice for ureteropelvic junction obstruction in selected cases, as it bears similar success rates and significantly lower morbidity compared to open pyeloplasty.[10,11] However, recent studies have shown that results of endopyelotomy deteriorate on long term and failures can be seen even after 2 years.[12,13] The percutaneous approach provides excellent visualization of the stenosed segment during the incision. Furthermore, new methods are currently being devised using endoscopic suturing techniques in conjunction with endopyelotomy, which further improve the success rate of the percutaneous procedure.[14]

Laparoscopic nondismembered pyeloplasty is principally same as with endopyelotomy. Neither diseased segment is excised nor reduction of pelvis done in both the procedures. In nondismembered pyeloplasty the incised area is sutured whereas in endopyelotomy it heals in non-obstructing fashion around a stent, so both the procedures are based on the same principle.

It is known that the success rate of endopyelotomy drops significantly with high grades of hydronephrosis. Van Cangh *et al*. reported 81% success rate in cases with low grade hydronephrosis and 60% in cases with high grade of hydronephrosis.[10] Danuser *et al*. found 87% success when pelvicalyceal volume was less than 50 ml and success worsened in patients with renal pelvic volume greater than 50 ml (76%).[15] We therefore excluded all cases with renal pelvic volume more than 50 ml to eliminate the bias of this factor in either group.

The anterior crossing vessels were found in one failed case of endopyelotomy group. In laparoscopy group, overall incidence of crossing vessels was 19.6% (36/184). The reported incidence of crossing vessels in the literature is ranging from 38 to 79% detected by spiral computerized tomography and endoluminal ultrasound.[16,17] Van Cangh *et al*. reported that its presence decreases success rate of endopyelotomy from 86 to 42 %.[10]

Other poor prognostic factors for success of endopyelotomy are redundant renal pelvis, high insertion of ureter, stricture length greater than 2 cm, renal ptosis with ureteral kinking, concomitant obstructing renal stones and glomerular filtration rate less than 25 ml/min.[18,19] However, there were no such cases in our cohort of patients.

Present study did not show any difference in success rate of both groups (*P* = 0.776). Klinger *et al*. reported success of nondismembered pyeloplasty to be 73.3% (11/15) which was similar to open nondismembered surgery.[8] Similarly Janetschek *et al*. reported 98% success rate of Fenger's pyeloplasty.[20]

Compared to laparoscopic nondismembered pyeloplasty, the operative time was significantly less in endopyelotomy group (*P*<0.01). This difference could be because of laparoscopic pyeloplasty being a technically demanding procedure; especially intracorporeal suturing and that most of these nondismembered pyeloplasty were done in early experience of the operating surgeons.

Concomitant pyelolithotomy was done in 3 patients in laparoscopy group and in 1 patient in endopyelotomy group. Renal stones can be removed by both modalities. Ramakumar *et al*. reported 90% stone free rate during concomitant pyelolithotomy and laparoscopic pyeloplasty.[21] However, percutaneous management is ideal when the UPJ obstruction is associated with upper tract stone disease as stones can be managed concomitantly.[22]

## CONCLUSION

In patients with UPJ obstruction with low volume pelvis and good renal function, endopyelotomy is as effective as laparoscopic nondismembered pyeloplasty with significantly shorter operative time. It may be preferred modality of treatment in these patients.
